# An Association of Serotonin with Pain Disorders and Its Modulation by Estrogens

**DOI:** 10.3390/ijms20225729

**Published:** 2019-11-15

**Authors:** Stephania Paredes, Santiago Cantillo, Kenneth D. Candido, Nebojsa Nick Knezevic

**Affiliations:** 1 Department of Anesthesiology, Advocate Illinois Masonic Medical Center, 836 W. Wellington Ave. Suite 4815, Chicago, IL 60657, USA; stephania.paredes@gmail.com (S.P.); tiagocantillo@hotmail.com (S.C.); kdcandido1@gmail.com (K.D.C.); 2 Department of Anesthesiology, University of Illinois, Chicago, IL 60612, USA; 3 Department of Surgery, University of Illinois, Chicago, IL 60612, USA

**Keywords:** serotonin, ovarian steroids, pain disorders, hormones, irritable bowel syndrome, migraine, headache, fibromyalgia, chronic fatigue syndrome

## Abstract

Ovarian hormones play an important role in pain perception, and are responsible, at least in part, for the pain threshold differences between the sexes. Modulation of pain and its perception are mediated by neurochemical changes in several pathways, affecting both the central and peripheral nervous systems. One of the most studied neurotransmitters related to pain disorders is serotonin. Estrogen can modify serotonin synthesis and metabolism, promoting a general increase in its tonic effects. Studies evaluating the relationship between serotonin and disorders such as irritable bowel syndrome, fibromyalgia, migraine, and other types of headache suggest a clear impact of this neurotransmitter, thereby increasing the interest in serotonin as a possible future therapeutic target. This literature review describes the importance of substances such as serotonin and ovarian hormones in pain perception and illustrates the relationship between those two, and their direct influence on the presentation of the aforementioned pain-related conditions. Additionally, we review the pathways and receptors implicated in each disorder. Finally, the objective was to stimulate future pharmacological research to experimentally evaluate the potential of serotonin modulators and ovarian hormones as therapeutic agents to regulate pain in specific subpopulations.

## 1. Introduction

Pain is subjective by definition and the treatment of pain is complex since its perception is influenced by neurobiological and psychological factors as well as by social-cultural differences and the hormonal behavior of each individual [1]. It is crucial to understand the causes, pathophysiology, and different targets of therapies of pain as pain is highly prevalent in the global population. Although the exact prevalence of pain is unknown, the prevalence of chronic pain among adults in the United States ranges from 11% to 40% with a wide subgroup variation [2,3]. Based on the analysis of the morbidity and mortality weekly report of the Center for Disease and Prevention Control (CDC) in 2016, an estimated 20.4% of adults in the U.S suffered from chronic pain and among these, 8% had high-impact chronic pain [2]. Chronic pain is also one of the principal reasons adults seek medical care; is the cause of a high disability index, and is closely linked with the opioid abuse epidemic [2]. The impact of chronic pain affects not only the individual’s quality of life, but is also responsible for the increase of disability-adjusted life years (DALY), a negative marker of overall health and life expectancy in the society [4,5,6,7].

Several neurotransmitters are implicated in the transmission and regulation of pain [8]. The role of each neurochemical and its association with specific types of pain has been well studied and described. Additionally, it is also known that important differences in pain sensitivity are due to variations of sex hormones. Some studies have demonstrated the direct stimulation and effects of these hormones on specific neurotransmitters, thus partially explaining the gender differences in the perception of pain conditions and the therapeutic responses of interventional therapies [9,10,11].

Serotonin is a monoamine found in both the central nervous system (CNS) and in the periphery. It is associated with several physiologic processes, and its dysregulations are in some measure involved in the pathophysiology of numerous well-known painful conditions such as irritable bowel syndrome, migraine headache, and primary non-migraine headache [8]. The objective of this literature review is to describe the relationship between serotonin and pain experience and how ovarian hormones can modify this relationship.

## 2. Serotonin Synthesis and Effects

Serotonin is synthesized from the dietary amino acid tryptophan by sequential hydroxylation and decarboxylation, and is then stored in presynaptic vesicles in neurons [8]. When a neuron is stimulated, the nerve terminals release serotonin, which initiates its effects by binding to a variety of receptors [8,12]. Serotonergic cells are found in the cardiovascular system, red blood cells, and the central and peripheral nervous system. In the central nervous system, they are mainly located on the brainstem in the raphe nuclei and project to the majority of the brain including the midbrain, prefrontal, parietal and occipital cortical regions, hippocampus, cingulate cortex, thalamus, cerebellum, and spinal cord [13].

The 5-HT receptors are divided into seven subfamilies (5-HT_1-7_) and 15 receptor subtypes. The role of each receptor and its association with pain conditions are not known with any certainty, and experimental studies with both agonists and antagonists have shown inconsistent results, possibly due to poor selectivity of the ligands as well as the diversity of the studied conditions [14,15,16]. Serotonin (5-HT) acts as an important neuromodulator with both nociceptive and antinociceptive effects. The specific effect depends on the type of receptor, amount of substance, and anatomical region where the action takes place. For example, the 5-HT_1A_ receptor in the CNS of rat model studies is associated with pain inhibition (antinociception) within the spine, while peripheral 5-HT can be responsible for the increase in the inflammatory response, thus exerting a nociceptive effect [9,17].

The spinal cord pain pathways and their interactions with 5-HT receptors are important in pain perception. Within the spinal cord, the descending pain modulatory pathways can exert both an inhibition (descending inhibition) or a facilitation (descending facilitation) type of influence on spinal cord nociceptive information processing, and this influence also depends on the type of receptor where neurotransmitters are acting. Although the exact receptor types implicated in spinal cord pain modulation are not completely understood, studies have shown the presence of at least four types of receptors (5-HT_1_, 5-HT_2_, 5-HT_3_, and 5-HT_7_) and several subtypes that can have an influence on these pain pathways [18,19].

For instance, Bardin L et al. demonstrated in their experimental study that receptor antagonists of 5-HT_3_ and 5-HT_2c_ could produce a total inhibition of the 5-HT anti-nociceptive effects. Additionally, there was no involvement of spinal 5-HT_1A_ and 5-HT_4_ receptors in spine pain modulation [14]. A different study showed that activation of the 5-HT_3_ receptors in the spine can inhibit nociceptive sensation, while its blockade potentiates the response by activation of the GABAergic interneuron system [20]. In general, the majority of the studies showed that the serotonin response depends on multiple factors such as the pharmacodynamics of the drug (serotonin receptor antagonist or agonist), the participation of specific receptors, and the duration of the stimulus being able to exert both an inhibitory or excitatory pain stimulus, or even maintain it if already provoked [8].

Serotonin has been implicated in several other neurophysiologic processes including the development of nausea when acting in the area postrema, in a group of neurons sensitive to humoral factors dominating in the chemoreceptor trigger zone, found in the floor of the fourth ventricle. Additionally, there are also roles in mood changes and cravings when acting in the hypothalamus; muscle spasms if stimulating the upper cervical spine; and increased transmission to cortical centers from the thalamus, which may be interpreted as pain [11,21].

## 3. Sex Hormone Influence on Serotonin

Sex hormones are steroid hormones synthesized in both the adrenal glands and gonads, which are derived from cholesterol by modification of the side group of steroids [1,22]. These hormones induce an effect on multiple organs including the central and peripheral nervous systems [10,23]. Additionally, it is known that mainly ovarian hormones have an important effect on a variety of neurotransmitters such as serotonin, the noradrenergic system, glutamate, gamma-aminobutyric acid (GABA), and the opioid system [13,24,25,26,27].

**Figure 1:**
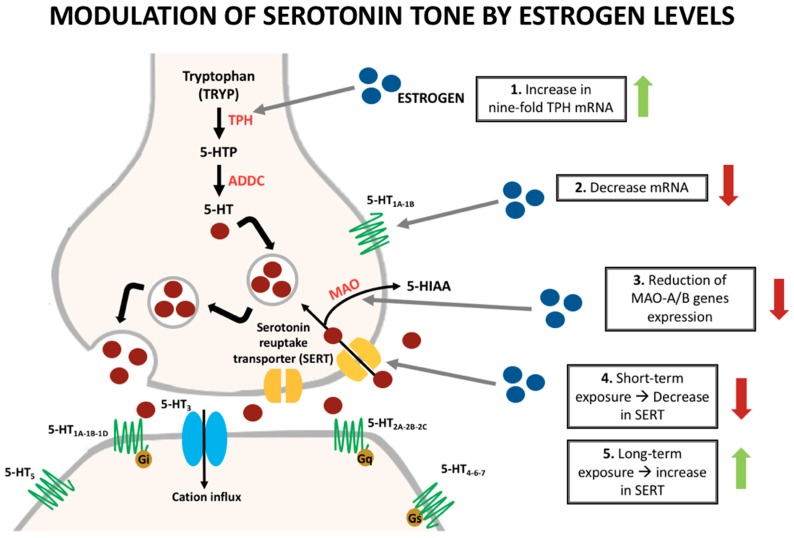
Modulation of serotonin synthesis and metabolism by estrogens.

Regarding the serotoninergic system, it is known that sex hormones can influence their synthesis, reuptake, and degradation (Figure 1). Pecins-Thompson et al. treated oophorectomized and hysterectomized monkeys with estrogen and estrogen/progesterone to compare the expression of important synthesis and metabolism of serotonin enzymes with a control group. In the treatment group, they found a nine-fold increase in tryptophan hydroxylase (TPH) mRNA, the rate-limiting enzyme in the synthesis of serotonin [28]. Furthermore, short-term treatment (28 days) showed a decrease in the amount of serotonin reuptake transporter (SERT). On the other hand, longer treatment (five months) caused an increase in the amount of the transporter [29]. According to more recent data, the influence of estrogen in the expression of the SERT depends on the duration of this hormone exposure [13].

Monoamine oxidases (MAO) A and B are the main serotonin degradation enzymes. An experiment suggested a reduction in the gene expression of MAO-A, the main degradation enzyme within the dorsal raphe and some hypothalamic nuclei in estrogen treatment groups when compared with controls. Furthermore, a reduction in the gene expression of MAO-B was also identified in the treatment groups, but only in the hypothalamic nuclei [13,24]. Some studies have also elucidated a modulation of the serotonin receptors with the influence of estrogen [30,31].

The 5-HT_1A_ receptors are mostly somatodendritic autoreceptors that regulate serotonin firing and can also alter the synthesis and release of the neurotransmitter. A study evidenced a decrease in the mRNA of the 5-HT_1A_ receptor mainly in the dorsal raphe nuclei in monkeys treated with ovarian hormones [30]. Additionally, a positron emission tomography (PET) scan study in postmenopausal women showed an increase by 21% on the binding of altanserin (selective 5-HT_2A_ ligand) to the receptors when women were treated with transdermal estrogen and micronized progesterone [31]. Studies in humans and non-human primates showed that areas involved in pain modulation such as the raphe nucleus, cingulate cortex, insula, prefrontal cortex, and amygdala possess a high density of 5-HT_1A_ receptors [32,33,34]. Furthermore, experimental research has demonstrated that 5-HT_1A_ receptor mimics the non-selective antinociceptive effects of serotonin, while on the other hand, 5-HT_1B_ receptors mimic the selective effect [8,35,36]. Other 5-HT_2_, 5-HT_3_, and 5-HT_7_ receptors are thought to intervene in the CNS modulation of pain. However, more studies are necessary to clarify their specific actions [8].

In general, studies support an increase in the serotonin tone in animal models when estrogen levels are higher when compared with the controls. However, this can be influenced by the length of the duration of estrogen exposure. In contrast, long-term therapy can also decrease serotonin tone as described in one study [29]. Therefore, multiple factors have to be considered to create a final hypothesis.

Although estrogen has multiple receptors, and the main effects are secondary to the stimulation of intra-nuclear genomic activation, it is believed that its influence on the modulation of serotonin’s action in pain is due to non-genomic or extracellular estrogen receptors (ER) [9]. This hypothesis is supported by experiments that confirm the presence of non-genomic receptors due to a rapid (10 min) effect in the change of pain perception. These receptors are localized outside the nucleus including the cytoplasm and cell membrane as G-protein coupled receptors (GPCRs) [37].

Although the evidence is based on experiments in non-human species, estrogen receptors (genomic and non-genomic) such as ERα, ERβ, GPR30 (a G-couple protein receptor), and serotonin receptors (5-HT_1-7_) are found in several mammals including humans [37,38]. Additionally, the serotonin metabolism system including its enzymes and transporters seems to be the same among different species; thus, it can be assumed that these changes and associations between serotonin and ovarian hormones can also occur inside the human body and in this way, the findings of these non-human studies could be extrapolated to humans.

In pain, estrogen has an important role in the modulation of the central serotonergic system. Some authors have hypothesized that the 5-HT_2A_ receptor plays an important role, and that administration of 5-HT_2A_ receptor antagonists would decrease the estrogen induced pain relief effect [39,40]. High concentrations of estrogen promote an increase in estrogen receptor (ER)β and downregulation of ERα. In general, ERβ upregulates the effect of 5-HT_2A_ activation, while ERα increases the effect of 5-HT_1A_ via nuclear factor kappa B (NFkB) [41]. Therefore, a high concentration of estrogen increases the density of 5-HT_2A_ receptors, which stimulates intracellular calcium (Ca) release and protein kinase C (PKC) activation, with cell-dependent effects. One common effect of the PKC is the negative feedback and uncoupling of the 5-HT_1A_ auto-receptors, decreasing the amount and effect of these receptors, and increasing the serotonin concentration through the inability of the system to produce negative feedback [42,43]. This specific modification in the pathway promotes vasodilation and has been associated with migraine and other headaches.

Some diseases and their relationship to alterations in neurotransmitters such as serotonin have been widely described. Currently, the modulation of these substances is one of the targets of management for these conditions and is a research topic of interest. Irritable bowel syndrome (IBS), migraine headaches, and other types of non-migraine headaches are part of well-known serotonin-associated conditions. In the next section, we describe the available evidence regarding these relationships.

## 4. Irritable Bowel Syndrome (IBS)

Irritable bowel syndrome is a functional bowel disorder, and its diagnosis depends on the presence of certain symptoms and the absence of organic or structural causes of these symptoms [44,45,46]. There is an estimated prevalence in Europe and North America of 10–15% and a female predominance with a ratio of 2:1 [47]. The pathophysiology is related to a wide range of factors such as motility and sensory abnormalities, increased painful thresholds, and a psychological component of the disease. Consequently, the treatment of this condition is a challenge, with no current universal standard treatment [48].

Rome IV criteria: Diagnostic criteria to irritable bowel syndrome (IBS)

Recurrent abdominal pain, on average, at least one day per week in the last three months, associated with two or more of the following criteria [44,45,46]:

(1) Related to defecation;
(2) Associated with a change in stool frequency;
(3) Associated with a change in stool form (appearance).

**Figure 2:**
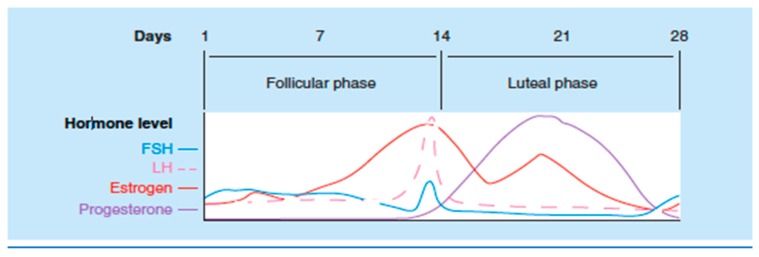
Relationship of steroid and gonadotropin hormones during the normal menstrual cycle. Adapted from *Pain and sex hormones: a review of current understanding* [1].

Variations in sex hormones have been implicated in changes in the presentation of the disease. Studies have demonstrated an increase of symptoms in certain phases of the menstrual cycle, mainly during the luteal phase and menses (Figure 2) [49]. The presence of estrogen receptors along the gastrointestinal tract, and its influence in the release of pain mediating substances such as serotonin, which plays an essential role in IBS pain perception, could explain this variation [50,51].

Polymorphisms of serotonin receptors influence the disease behavior, mainly in the 5-HT_3_ receptor, which has an important role in the visceral pro-nociceptive pathway. Alterations in the A and E subunits of 5-HT_3_ are related to increased IBS/diarrhea risk. Furthermore, there is an increase in the density of the receptors due to possible polymorphisms in the upregulation mechanisms [52,53,54]. Some studies have shown that spinal 5-HT_3_ receptor activation increases visceral pain transmission by the release of substances such as substance P, calcitonin gene-related peptide, and neurokinin A from primary afferent nerves [55].

Ovarian hormones have a clear influence on visceral sensitivity, not only through serotonergic pathways, but also by mast cell regulation and modulation in the stress response [50]. Studies have demonstrated that the presence of estradiol and progesterone receptors in mast cells and their binding to estrogen triggers the degranulation, increasing the presence of the inflammatory substance and, in the same way, the visceral sensitivity [40,56,57,58]. Additionally, estrogen effects cortisol’s receptors along the enteric neurons during the stress response, causing an increase in visceral sensitivity [50].

Some experiments have demonstrated higher levels of serotonin synthesis and its receptor in the brains of rats that present with visceral pain such as IBS. Moreover, there is a clear effect of estrogens in several studies in the modulation of the levels of serotonin in the nervous system and indirectly on the level of pain [9,17]. Additionally, despite the demonstrated improvement of pain in IBS with a serotonin antagonist drug, it is also known that the response to treatment is different and is influenced by gender, with an enhanced response in women than in men, therefore supporting the theory of serotonin modulation by estrogens [59].

Alosetron is a selective 5-HT_3_ receptor antagonist that is the only FDA (US Food and Drug Administration)-approved drug for IBS. Studies have demonstrated more efficacy in women with diarrhea-type predominance IBS [60,61]. These disparities could be explained by differences in drug metabolism by CYP2C19 between the sexes, SERT gene polymorphisms, or limbic system activation with higher limbic activity in women during pain production [62,63,64]. The exact mechanism of action of alosetron is unknown; however, studies have demonstrated an inhibition of expression of c-Fos genes, which are related to pain generation, and suggest that this drug can exert its effects at a spinal level to block the visceral afferent nociceptive signaling [65].

Experiments demonstrate an enhanced 5HT receptor expression during the late luteal phase when estrogen and progesterone levels are decreased, and this was also associated with an increase in symptoms and visceral sensitivity [40]. These findings probably justify the epidemiological differences in the prevalence of some pain conditions depending on hormonal profiles, as the increase of IBS with menses or higher prevalence of pain in postmenopausal women, in which the hormone levels are low [59,66,67].

The understanding of visceral pain pathways is important in recognizing the serotonin relation and influence of it in pain modulation in IBS. Pain regulation pathways in visceral pain include vagal and spinal afferents that project into the CNS, both facilitating and/or inhibiting the sensory transmission to the spinal cord. The cell bodies of the vagal afferents are in the nodose ganglion, while the cell bodies of the spinal afferents are found in the dorsal root ganglia [68].

Specifically, in visceral pain, there is an antinociceptive action of estrogen on the serotoninergic system due to afferent-driven vagal inhibition of the pain. On the other hand, the pro-nociceptive action occurs because of the enhancement of serotonin secretion in the intestinal mucosal mast cells (IMMCs); cells in which estrogen receptors have been found and that have been associated with its degranulation, increasing the visceral motor response and spinal or supraspinal processing of visceral nociception, mainly in IBS [9]. Furthermore, as described previously, the interference of serotonin in pain modulation depends on the receptors, route of administration, type of pain, and influence of other substances on the release of this neurotransmitter [8].

Therefore, despite the complex relationship between estrogen and serotonin in pain modulation in patients with IBS, it seems that the importance of the site of action of serotonin determines the effects in pain modulation. The increase in serotonin levels in the periphery is responsible for the nociceptive action through estrogen receptor activation in the intestinal mucosal mast cells, and also the activation of spinal receptors such as 5-HT_3_. On the other hand, increases in serotonin levels in the vagal afferent pathways are related to being antinociceptive. Finally, estrogen levels seem to be closely related to the modulation of serotonin in IBS, with a direct relation in the periphery (an increase of serotonin due to IMMC degranulation) and alteration of the receptors’ expression when there is an estrogen withdrawal phase, causing more pain.

## 5. Migraine

Migraine is two to three times more common in women than in men, especially during childbearing ages [11,69]. Two subclassifications of migraine—pure menstrual migraine without aura and menstrually related migraine without aura—are included in the 2018 International Classification of Headache, and are closely related to hormone alterations, especially estrogen withdrawal during the menstrual cycle. The importance of this classification is the confirmed effectiveness of hormone prophylaxis in these entities when compared with other types of migraines [70]. It is also recognized that the incidence of migraine crises is the highest two days before to three days after the start of menstruation [71,72,73].

Vasodilation in the brain may be the cause of the headache [74]. The activation of the 5-HT_1B_ receptor induces vasodilation, normally balanced by the activation of the 5-HT_A2_ receptor, which leads to vasoconstriction. Unlike the 5-HT_1A_ receptor, 5-HT_1B_ is not downregulated or uncoupled by estrogen. Therefore, the estrogen-induced increase in serotonin results in vasodilation without the capacity to appropriately compensate using the 5-HT_2A_ receptor [40,75].

Additionally, it is believed that trigeminal neurons are hyper-sensitized during this disease, increasing pro-nociceptive substance expression as calcitonin gene-related peptide (CGRP) and transient vanilloid receptor 1 (TrpV1), a nonselective capsaicin-binding cation channel [76,77,78]. Hormone fluctuations may change the expression level of those peptides, and newer studies have shown an inverse correlation between estrogens and CGRP, which may explain the peri-menstrual sensitization of the trigeminovascular system. Sex hormones can have an important role in modifying the c-Fos protein pathway, regulating gene transcription, and causing an increase in the transmission of neurotransmitters such as serotonin and norepinephrine [11]. These neurotransmitters are responsible for the development of neurogenic inflammation and vasodilation through the sensory afferents between intracranial vessels and the trigeminal system [79].

Serotonin plays an important role in migraine. An increase in its transmission in the central nervous system in patients with migraine and the induction of headache with serotonin agonists in experimental studies support this idea [11]. Some experiments have shown the effect on serotonin synthesis in trigeminal ganglion neurons due to local increases in the rate-limiting enzymes such as TPH, and demonstrate that this synthesis can be modulated by ovarian estrogens [80]. However, the influence and effects of serotonin in the sensory system are complex, modulating the activity of a variety of receptors including potassium channels, calcium channels, tetrodotoxin-resistant sodium channels, TRPV1, and intracellular calcium, which in conjunction regulate the excitability of the sensory neurons [80,81,82,83,84,85].

In women experiencing migraine, PET scan imaging has demonstrated an increase in the synthesis of serotonin in all regions of the brain [13,86]. Furthermore, both serotonin and opioid systems are thought to be essential parts of the inhibitory neurotransmitter systems for the trigeminal pain pathways [87]. On the other hand, substances that increase peripheral serotonin can relieve headache due to an auto-inhibitory effect in central serotonin production [11].

Ovarian hormones such as estrogen can influence the presence and intensity of the symptoms. Currently, there is a bimodal theory in which either abrupt decreases in estrogen levels or a chronically high plasma estrogen concentration modulates the trigeminal pain pathway [88]. However, clinical observations have shown an improvement in headaches with the increase in estrogen or in the absence of estrogen withdrawal (pregnancy, menopause), and a worsening of migraine with estrogen withdrawal (menses, postpartum, and use of oral contraceptives) [11].

In patients with menstrual-related migraine, the activation of ER-α receptors also regulates other modulatory pathways, increasing the activity of nitric oxide synthase in endothelial cells, especially in the luteal phase (estrogen withdrawal) and the L-arginine pathway, and these were related to an increase in migraine crisis [89].

Concerning the relationship between serotonin and sex hormones in this condition, a small clinical study comprised of 10 women with and six without status migrainosus were followed during one month of combined hormonal contraception therapy (21 days of hormones and seven days free of hormones) associated with a serotonin agonist. During the placebo week, the women were divided into either the transdermal estrogen or placebo group. In the placebo group, the results demonstrated significant impairment in the neuroendocrine system, with a decrease in cortisol levels and alterations of prolactin secretion worsening the migraine attacks. However, once the patients received both an estrogen and serotonin agonist, a clinical improvement was seen [90].

In patients with conditions such as chronic migraine or menstrual status migrainosus, Cassidy et al. found a reduction in 5-HT_1A_ receptors during the early follicular phase, when the estrogen levels are low [91]. Additionally, the use of a serotonergic agonist such as sumatriptan has proven to be effective in the treatment of acute migraine, probably explained by the hypothesis of the auto-inhibition of central serotonin level production [90].

Finally, the influence of sex hormones in the serotonergic system and the pathophysiology of migraine is complex, and currently, a bimodal theory exists. The effects of serotonin in migraine are not only influenced by the level of the substance, but also by receptor subtypes, and by other pro- or antinociceptive substances present in the environment such as CGRP, histamine, and bradykinin [80]. However, considering the latest factors such as menstrual cycle phase, the velocity of changes in hormones levels, and the specific site of action (central or peripheral of serotonin), the behavior of migraine attacks and the response to therapies could be expected.

## 6. Primary Non-Migraine Headache

Regarding other types of headache, less information is available about the influence of both estrogen and serotonin. However, the existing studies show that estrogen probably upregulates serotonin [11]. Silberstein et al. also demonstrated that estrogen withdrawal decreases peripheral serotonin, and this leads to a reduction in the self-inhibitory effect of the 5-HT receptors, which in theory results in an increase in serotonin levels, and has been associated with increases in the intensity of pain [92,93].

Tension-type headache (TTH) is the most prevalent primary headache worldwide, with a lifetime prevalence of 69% in men and 88% in women [94]. In surveys, no difference in children’s prevalence was found; however, the female incidence started to increase after puberty when estrogen levels increase [95,96].

Concerning the peripheral factors implicated in the physiopathology of TTH, activation of nociceptors increases the tenderness of pericranial myofascial tissues to palpation, and has a direct relationship to the intensity of the attacks [97,98]. This nociceptor sensitization could be induced by a variety of substances including serotonin, which creates an inflammatory environment, and estrogen, which acts as an important upregulator of serotonin synthesis [22].

In retrospective surveys, 38% to 46% of women reported an increase in their headache with menses when estrogen withdrawal occurred [99,100]. There is a tightly described relationship between menses and the increase of TTH episodes. Additionally, as opposed to migraine, it seems that there is no improvement after menopause in TTH patients, but worsening is possible [22].

On the other hand, cluster headache (CH) is the only primary headache with a higher prevalence in men [101]. When it does affect women, it is more common after menopause when the estrogen levels are lower. This supports an estrogen protective effect hypothesis; however, the basis behind this protective mechanism is still unclear [102,103].

The activation of a trigeminal autonomic reflex is responsible for the pain of cluster headaches, and elevated levels of substances such as CGRP are found in the trigeminal ganglion cell bodies, which innervate the blood vessels [104,105]. The hypothalamus and its connection with trigeminal neurons can also be important regulators of this disease [106].

## 7. Fibromyalgia

Fibromyalgia is the most frequent cause of chronic widespread muscular pain, and is often associated with somatic symptoms, fatigue, cognitive disturbances, and sleep problems [107,108]. The clinical diagnosis is based on the presence of generalized pain in at least four of five regions for at least three months and a widespread pain index (WPI) ≥7 and symptom severity scale (SSS) score ≥5, or WPI of 4-6 and SSS score ≥9 [109,110,111]. The global prevalence is approximately 2–3% and increases with age. This prevalence is six-times more frequent among women, being their most common cause of pain between 20 and 55 years [112,113,114].

The etiology and pathophysiology are still unclear. Although controversial, some studies suggest that central sensitization, a disorder in pain regulation, could be the principal cause of this disorder [115,116]. Experimental models suggest that attenuation in the function in descending pathways increases pain sensitivity and response of these patients [117,118,119]. Multiple neurobiological alterations are also associated with this disorder including the hypothalamic-pituitary-adrenal (HPA) axis, especially an exaggerated adrenocorticotropin hormone (ACTH) response to corticotropin-releasing hormone (CRH) [120]. Additionally, serotonin and norepinephrine, two key neuromodulators in the transmission of pain in the descending nociceptive modulatory pathways in both the brain and spinal cord, have been associated with this condition [121]. Other diseases have been related with fibromyalgia, and are thought to share a similar pathophysiologic mechanism such as irritable bowel syndrome (IBS), and migraine [116].

Serotonergic dysfunction is one major hypothesis of fibromyalgia, which is supported by the efficacy of drugs that alter serotonin metabolism [114,122]. Some reports in patients with fibromyalgia mention a low serotonin level; however, this reduction could be associated with several factors including altered hypothalamic pituitary adrenal axis (HPA) and sympathetic hyperactivity [123]. The low serotonin (5-HT) synthesis level could be associated with a reduction in the levels of melatonin, which partially explain these patient’s disturbed sleep patterns.

Some studies have considered that estrogen deficiency could be not only a promoting factor of this condition, but also the reason why it is more prevalent in women [112]. During experimental evaluations, the symptoms are usually more prominent in certain sex-related events such as premenstrual periods, pregnancy, and menopause. Although this association is also controversial and a clear susceptibility given by a hormonal profile has not been demonstrated in experiments, the absence of ovarian hormones is at least associated with increased nociceptive sensation [124].

It is known that gonadal steroids such as estrogen affect the glucocorticoid negative feedback loop, and also impair the shut-off of ACTH and corticosterone secretion when a person is exposed to a stressful situation, leading to enhanced activity of the HPA axis. For instance, the reduction of estrogen stimulation during menopause is related to a relative hypofunction of the HPA axis [125]. A prospective study evaluated pain sensation in patients with fibromyalgia vs. controls; groups were divided into patients with early menopause (less than 49 years) or late age-onset menopause (older than 49). Patients with early-onset menopause showed a higher index of pain and non-pain sensitivity than women with late-onset menopause. The author concluded that a shorter exposure time to estrogens could influence the pain hypersensitivity of patients with fibromyalgia [112].

There is no standard treatment for patients with fibromyalgia, a combination of several pharmacologic and non-pharmacologic treatments is recommended [126,127,128,129]. Antidepressants like amitriptyline, selective serotonin, and norepinephrine reuptake inhibitors (SNRI) such as duloxetine and milnacipran, and the anticonvulsant pregabalin are approved by the FDA for this condition, and randomized clinical trials have proven their efficacy in the reduction of symptoms and also the improvement in life quality [108,128,129,130,131,132]. A systematic review and meta-analysis comparing duloxetine vs. the placebo for the reduction of pain in fibromyalgia evaluated 2249 patients and demonstrated that duloxetine was significantly more likely than the placebo to reduce pain by at least 50% at 12 weeks, and at 28 weeks [133]. Additionally, long-term benefits were shown in multiple experiments including a multicenter, randomized, double-blind, placebo-control trial that contained 520 patients, where duloxetine reduced the pain severity significantly at three and six-months [134]. Additionally, duloxetine was shown to be safer and more effective during an average duration of one year of follow-up [135]. This evidence supports the essential role of serotonin in fibromyalgia pathophysiology.

Non-pharmacologic management includes exercise, cognitive behavioral therapy, patient education, and acupuncture. The mechanism of action of acupuncture is unknown; however, it is thought that stimulation of acupoints can alter the concentration of some pain mediators including endorphin, substance P, encephalin, and serotonin in the brain and local tissues [136,137]. The effect of acupuncture has been evaluated in controlled clinical trials. Sprott et al. evaluated the pain level assessed with VAS scores and the changes in the serum levels of serotonin and substance P after six acupuncture treatments. Their study demonstrated not only a significant improvement in pain scores, but also a decrease in platelet serotonin levels, and an increase in the serum serotonin and substance P [137]. In a different study, patients who underwent acupuncture treatment also demonstrated a reduction in pain and a significant increase in serotonin levels.

Although the previous experiments support the role of serotonin with pain modulation in fibromyalgia, the exact association remains unknown and controversial due to literature supporting the opposite idea [136]. There is an association of estrogen with the disease, which in part could explain the predominance among women. Additionally, serotonin seems to play a role in the pathophysiology of fibromyalgia. However, no association between the three of them has been widely explored in the literature.

## 8. Chronic Fatigue Syndrome

Additionally known as myalgic encephalomyelitis, chronic fatigue syndrome (CFS) is a condition characterized by medically unexplained severe and prolonged fatigue with an impact on the quality of life [138,139,140,141,142]. Although the etiology is the subject of some controversy and seems to involve multiple neuronal, endocrine, and immune alterations, specific causes still remain unclear [143]. Recently, the role of several humoral systems has been studied including the glutaminergic and serotonergic pathways. However, neurotransmitter abnormalities seem to only partially cause the syndrome since other metabolic abnormalities including glycogen, carnitine, and fatty acid disturbances have been shown to impact the disease [143]. Similarly, oxidative stress, infections, autoantibodies, and functional/structural abnormalities are thought to play a role in the pathogenesis of CFS (i.e., superoxide dismutase abnormalities, immune deregulation, DNA methylation patterns, mitochondrial dysfunction, among others) [144,145,146,147,148].

Although some authors have also explored the association between sex hormones and CFS, to the best of our knowledge, no study has been performed to assess the relationship between 5-HT and sex hormones in the pathogenesis of CFS.

Studies in animal models suggest an interaction between the immune system and the serotonergic pathway. Upon artificially-induced immune system activation, microglia, an essential immune cell present in the CNS, releases the pro-inflammatory cytokine IL-1β [149,150]. Astrocytes, another CNS cell responsible for regulating the amount of neurotransmitters available for neuronal activity, highly expressed concentrations of IL-1β receptors, and its activation have been proven to increase the expression of the serotonin transporter 5-HTT in specific glial cells [149]. It is hypothesized that increased uptake of serotonin by astrocytes leads to increased deactivation of serotonin by monoamine oxidase A, the enzyme responsible for deamination and scavenge of 5-HT for further re-utilization [151,152]. As a consequence, the interaction between microglia-astrocytes is responsible for a lower availability of serotonin to act on 5-HT receptors, that being especially important for the 5-HT1A receptor [153].

Potential therapeutic targets related to serotonin include antidepressants including imipramine (non-selective 5-HT reuptake inhibitor), a medication that has been shown to abolish the inflammatory-induced decrease in extracellular levels of serotonin, and which could potentially impact the pathology [150]. Additionally, specific agonists for the 5-HT1A receptors or inhibitors of 5-HTT may be promising medications for the treatment of CFS [143].

The role of hormones in the pathophysiology of CFS has also been explored. A study comparing blood levels of luteinizing hormone (LH), follicle-stimulating hormone (FSH), estradiol, progesterone, and cortisol in premenopausal females diagnosed with CFS and paired healthy controls found no significant difference between CFS and fatigue-free patients for LH, FSH, estrogens, and progesterone, although CFS-diagnosed patients were shown to have significantly lower levels of cortisol [154].

Veldman et al. [155] described a case of CFS in a patient with dysmenorrhea membranacea. This is an uncommon condition characterized by a spontaneous sloughing of a large piece of endometrium retaining the shape of the uterine cavity. This disease has been associated with high levels of progesterone, and in this specific case, the patient was exposed to contraceptive hormone therapy that included progestin and estrogen. Although the authors can merely suspect an association between CFS and hormone levels in this case, it is certainly interesting to note the resolution of the fatigue after discontinuation of the hormone therapy as well as the presence of inflammation on laboratory findings during the contraceptive treatment, which also resolved soon after the hormone was withdrawn [155].

In summary, the association between sex-hormones and CFS as well as the integration of both with the serotonergic pathway, remains unclear. Further research needs to be conducted to determine the potential of hormone-related therapeutic targets for the treatment of CFS.

## 9. Serotonin Modulating Analgesics

The role of serotonin in pain is supported by the fact that there is a high rate of comorbidity with other serotonergic dysfunctions such as mood disorders. Furthermore, medications with effects on serotonin were used off-label for pain due to the empirically observed improvement after initiation.

The serotonergic pathway is a therapeutic target for several chronic pain-related conditions like fibromyalgia and neuropathic pain [156]. For chronic pain management, tricyclic antidepressants are the most commonly used antidepressants. However, the efficacy of selective serotonin (SSRI) and serotonin-noradrenaline reuptake inhibitors (SNRI) to decrease pain and reduce the need for opioid therapy has been studied [156,157,158].

Duloxetine, a medication of the SNRI family, has been shown in controlled randomized trials to decrease pain in diabetic and non-diabetic peripheral neuropathy, osteoarthritis, fibromyalgia, in postoperative analgesia, pain in the orofacial region, and exceptionally for acute pain [157,159]. With almost no effect upon cholinergic and histaminergic receptors, duloxetine and other SNRIs are primarily indicated for depressive and anxiety disorders, despite the increase in available evidence supporting their use as an analgesic.

The pain-related condition where SNRIs have the most evidence is for neuropathic pain. It is thought that a nociceptive stimulus originating in the periphery is transported by primary sensory neurons through the dorsal horn of the spinal cord and then to the brain through an ascending pathway. There is also an antinociceptive system with descending fibers originating in the brainstem. These neurons release serotonin and noradrenaline as their neurotransmitters and it is thought that dysregulation in this descending tract may impede the pain-relief activity [158]. A recent meta-analysis of seven studies showed the superiority of duloxetine over placebo to decrease pain by at least 30% in patients with fibromyalgia [156].

Despite the wide use of SNRIs for neuropathic and chronic musculoskeletal pain, there is scant evidence that supports their use for other conditions mentioned in this paper. Some recent studies have suggested an important role of these drugs in the prevention of migraine. However, no literature has supported its use as a headache abortive therapy yet [160]. Newer evidence in favor of antidepressants as pain relieving agents is increasing. However, more data about effectiveness and tolerability need to be collected.

## 10. Discussion

Serotonin is an important neurotransmitter involved in multiple physiologic processes such as mood, sleep, and pain [8]. There is increasing evidence with experimental models and brain imaging supporting anti-nociceptive effects mediated by the 5-HT_1A_ and 5-HT_1B_ receptor [30,31]. However, the exact role of other serotonin receptors in pain modulation still remains unclear.

On the other hand, the interaction of the estrogen–serotonergic pathway seems to be mediated by the activation of genomic and non-genomic estrogen receptors, as cytoplasmic and membrane receptors. The 5-HT_2A_ receptor has shown to importantly decrease pain when estrogen levels are high, and its blockage by a 5-HT_2A_ antagonist tends to decrease the estrogen-induced pain release [39,40]. Estrogen receptors α and β respond differently to estrogen concentrations, with high hormone levels inducing an increased in the expression of ERβ compared with ERα [41]. This cycle promotes an increased tone in 5-HT_2A_ and uncoupling of the 5-HT1A with subsequent abolition of the negative feedback system [42].

Sex-hormones and serotonin have a wide variety of receptors distributed throughout the peripheral and central nervous system, each receptor located in specific cells and with different mechanisms of action [12,33,37]. As a consequence, there is a wide range of physiologic effects and potential pathophysiologic changes capable of causing, promoting, or precipitating pain-related conditions. The specific effects of the serotonin–estrogen interaction for every clinical condition is summarized in Table 1.

As mentioned previously, the research output on this topic is increasing, and some authors have published clinical trials comparing serotonergic and estrogen medications with other therapeutic options. Although more research still needs to be conducted, there are promising results about the efficacy of SSRIs and SNRIs for the treatment of several painful conditions including, but not limited to IBS, fibromyalgia, neuropathic pain, and migraine [161,162,163]. However, although estrogens seem to also have a role in the modulation of acute pain due to the location of receptors in the membrane and cytoplasm, there is scant literature exploring the efficacy of hormonal treatment to decrease pain intensity in acute conditions.

On the other hand, basic science studies elucidated the potential key role of the receptors 5-HT_1A_ and 5-HT_1B_ for pain modulation as well as the relevance of the 5-HT_2A_ in the interaction of both biological pathways, estrogens and serotonin, and their antinociceptive effects. However, little is still known about the pain-modulating effects of different serotonin receptors and their importance as therapeutic targets for the treatment of pain [14,18]. Similarly, the wide variety of mechanisms of actions and cell tropism of estrogens makes a complete understanding of the complete pathway difficult [42].

## 11. Conclusions

The association of serotonin with pain disorders and its modulation by estrogens has been well studied for certain conditions including irritable bowel syndrome and migraine, where a clear effect of those substances has been elucidated. However, for other pain conditions, it is not completely understood and further investigation is needed.

Evidence suggests an important role of endogenous sex hormones in the pathophysiology of IBS, migraine, fibromyalgia, and CFS, which could explain at least in part the increased prevalence in women. Additionally, the neurotransmitter serotonin is strongly associated with diseases such as fibromyalgia and IBS. In general, studies have demonstrated that estrogen withdrawal is associated with an increase in serotonin tone and thus painful conditions. This relationship between ovarian steroids and serotonin raises the question not only about the potential therapy options, but also about the treatment response differences between genders.

Finally, although the current evidence leads us to believe in a significant influence of estrogen in the modulation of serotonin in pain, this assumption arises from animal experiments, and human studies are needed to confirm this association.

## 12. Limitations

Unfortunately, evidence for several clinical conditions is still controversial, and there is almost no information about the relationship of serotonin and ovarian hormones for illnesses such as fibromyalgia, where sex-hormones have been proven to increase pain. There are still contradictory results for several conditions such as visceral pain, headache, fibromyalgia, and chronic fatigue. Few basic sciences studies have been performed in humans, and the role of serotonin must be overlapped from animal models when available. There is an increasing amount of clinical trials comparing the efficacy to decrease pain between different SSRIs and SNRIs and other interventions or placebo; although several authors have studied these medications for fibromyalgia [132], IBS [164], and migraine [163], no controlled trials have been performed for the other conditions discussed in this manuscript.

## Figures and Tables

**Table 1:** Serotonin and estrogen modulation of pain conditions.

Condition	What We know
Irritable Bowel Syndrome	- Increase of symptoms during luteal phase and menses - Increase levels of serotonin synthesis and its receptors - 5 HT_3_ receptor polymorphisms cause increased IBS/diarrhea risk - 5-HT_3_ receptor activation induces release of substance P, CGRP, and neurokinin - The interference of serotonin in pain modulation depends on the receptors, route of administration, and type of pain: ✓ Antinociceptive action of estrogen on the serotoninergic system due to afferent-driven vagal inhibition of pain ✓ The increase in serotonin levels in the periphery caused by the activation of estrogen receptors in IMMCs and spinal receptors is pro-nociceptive - During the estrogen withdrawal phase, there is an increase in pain perception in patients with IBS - Serotonin antagonist (Alosetron) has a demonstrated efficacy in IBS patients, with predominance in women
Migraine	- More common in women during childbearing ages - Estrogen causes activation of 5-HT_1B_ without the compensation of the 5-HT_2A_ receptor; this causes vasodilation and thus headache - Estrogen causes release of nociceptive substances including calcitonin gene-related peptide CGRP and TrpV1 - Estrogen can modify the c-Fos protein, which regulates the transcription of serotonin and norepinephrine - Estrogen increases the synthesis of serotonin by modulation of the rate-limiting enzyme TPH - Bimodal theory: an abrupt decrease in estrogen levels or a chronically high plasma estrogen concentration modulates the trigeminal pain pathway - Serotonin agonists such as Sumatriptan are effective for acute migraine
Primary Non-Migraine Headache	- Increase of headache female incidence after puberty - Estrogen upregulates serotonin - Estrogen withdrawal is associated with an increase in the intensity of pain - Activation of nociceptors induced by serotonin - Activation of trigeminal autonomic reflex is responsible for pain in cluster headaches
Fibromyalgia	- The prevalence is six-times more frequent among women - Serotonin and norepinephrine modulate the transmission of pain in the descending nociceptive modulatory pathways in the brain and spinal cord - Low serotonin synthesis level in patients with fibromyalgia - Estrogen deficiency as a promoter of the disease - Shorter exposure time to estrogens influences the pain hypersensitivity - Duloxetine and milnacipran, selective SNRIs, are FDA approved drugs for fibromyalgia
Chronic Fatigue Syndrome	- Role of glutaminergic and serotonergic pathways in the pathophysiology - Activation of IL-1β receptors in the disease, increases the expression of serotonin transporter (5-HTT) in glial cells - Reuptake increase of serotonin by astrocytes, increase the deactivation of serotonin by monoamine oxidase A in fibromyalgia patients - Imipramine (non-selective 5-HT reuptake inhibitor) has been used in this condition - Resolution of fatigue and decrease of inflammatory mediators in experiments in patients with CFS, after the discontinuation of hormone therapy

**Abbreviations:** IBS, Irritable bowel syndrome; IMMCs, Intestinal mucosal mast cells; CGRP, calcitonin gene-related peptide; TrpV1, transient vanilloid receptor 1; TPH, tryptophan hydroxylase; SNRI, serotonin and norepinephrine reuptake inhibitors; FDA, Food and Drug Administration; IL-1β, interleukin 1 beta; CFS, Chronic fatigue syndrome.
